# Rhino-orbital-cerebral mucormycosis in diabetic ketoacidosis: A classic clinical presentation still unknown in Senegal

**DOI:** 10.1016/j.idcr.2025.e02244

**Published:** 2025-05-04

**Authors:** Khadim Diongue, Sokhna Moumi Daffé, Abdourakhmane Sané, Maguette Ndoye, Mamadou Wagué Gueye, Mor Ngom, Papa Silman Diawara, Stéphane Ranque, Bécaye Fall

**Affiliations:** a Laboratory of Parasitology and Mycology, Aristide Le Dantec University Hospital, 30 Avenue Pasteur, Dakar 12900, Senegal; b Laboratory of Medical Biology, Hôpital Principal de Dakar, Route de la Corniche Estate, Dakar 12900, Senegal; c Intensive Care Unit, Hôpital Principal de Dakar, Route de la Corniche Estate, Dakar 12900, Senegal; d Laboratory of Parasitology and Mycology, Institut Hospitalo-Universitaire Méditerranée Infection, 19-21 Boulevard Jean Moulin, Marseille 13005, France

**Keywords:** Rhino-orbital-cerebral mucormycosis, Diabetic ketoacidosis, Rhizopus oryzae, Senegal

## Abstract

Rhino-orbital-cerebral mucormycosis (ROCM) is an invasive mycosis caused by fungi of the order Mucorales. It mainly affects patients with uncontrolled diabetes. This case report concerns a 27-year-old woman with diabetic ketoacidosis who presented with a large erythematous plaque localised in the right orbitofrontal region with exophthalmos and patches of necrosis that rapidly led to death within 3 days before the diagnosis of ROCM due to *Rhizopus oryzae*, the causal agent. So, without rapid diagnosis and swift management, ROCM results in a potential fatality.

## Introduction

Rhino-orbital-cerebral mucormycosis (ROCM) is an invasive fungal infection caused by fungi of the order *Mucorales* [1]. ROCM is a potentially fatal opportunistic infection which mainly affects immunocompromised patients or those with metabolic disorders, and/or uncontrolled diabetes [1], [2]. About 70 % of patients with ROCM have diabetes mellitus (DM), making it the major risk factor for ROCM [3]. Due to the lack of specific clinical symptoms, diagnosing mucormycosis is challenging in the early stages. However, if mucormycosis is suspected, urgent intervention is crucial as the infection has a high mortality rate due to its rapid and destructive progression [1]. Magnetic resonance imaging (MRI), head/orbit CT scan, and other endoscopic imaging are essential to determine intracranial and sinus spread in ROCM [4]. A final diagnosis of mucormycosis combines positive *Mucorales* culture and microscopic evidence [5]. For effective management of ROCM, liposomal amphotericin B-based antifungal therapy combined with aggressive surgical debridement and elimination of underlying risk factors is essential using a multidisciplinary approach [3].

Even though the incidence rate of this rare disease has risen in recent years, particularly during the COVID-19 pandemic [6], it is still unknown and rarely described in sub-Saharan Africa, where the diagnosis remains a challenge [2], [7]. This work reports a fatal case of ROCM due to *Rhizopus oryzae* in Dakar, Senegal.

## Case

On June 22, 2022, a 27-year-old female living in the suburbs of Dakar in Senegal, with no specific pathological history, was admitted to the emergency department of the *Hôpital Principal de Dakar*. The patient was referred from a health centre in Dakar for the management of diabetic ketoacidosis of inaugural origin (**day 0**). The symptomatology, reported by the parents, lasted for three days and was marked by the appearance of a non-traumatic swelling of the right eye on a background of chronic rhinitis (**day −3**).

**Fig. 1:**
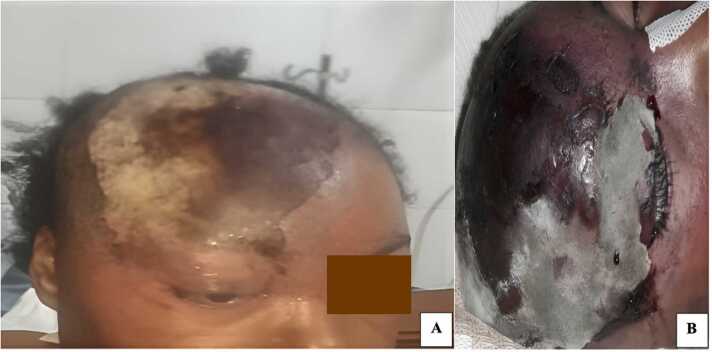
Pictures of the patient. Homolateral right exophthalmos and an erythematous right orbitofrontal placard with necrotic patches on admission (A). Extension of necrosis to the entire right frontal-parietal-temporal region, with visible fungus growth on the skin (B).

On admission, physical examination revealed an altered general condition in an obnubilated patient (Glasgow score: 13/15) with no fever (**day 1**). Emergency capillary glycemia was 3.27 g/L, with ketones on the urine dipstick (++). The patient's physical examination also revealed a large erythematous plaque localised in the right orbitofrontal region with right exophthalmos and patches of necrosis with sharp edges (Fig. 1A), as well as whitish deposits on the tongue and inner cheeks that can be removed with a tongue depressor. In addition, large linear stretch marks were noted on the thighs, the shoulders and the abdominal wall as well as the sub-mammary and inguinal folds intertrigos, and a brown foul-smelling discharge with a rotten fish odor in the vagina.

On the laboratory evaluation, white blood cell (WBC) counts showed reactive neutrophilic polynucleosis (WBC: 23 × 10^9^/L), microcytic hypochromic anaemia (Hb: 7.7 g/dL), positive C-reactive protein (CRP: 211 mg/L), hypokalemia (3.3 mmol/L), and elevated troponins (3.3 μg/L). HIV and hepatitis B serologies and malaria thick blood smear were negative. Urine cytobacteriology, liver, thyroid, renal function, and initial hemostasis tests were normal.

According to clinical symptoms and laboratory results, the patient was admitted to intensive care with the diagnosis of unbalanced diabetes of the ketoacidosis type, complicated by periorbital cellulitis. A rehydration and insulin therapy protocol associated with antibiotic therapy based on metronidazole (1.5 g/d) and amoxicillin + clavulanic acid (3 g/d) was instituted. Wound cleansing was prescribed, and necrosectomy was discussed. The initial treatment did not improve the patient's condition (**day 2**). The clinical picture deteriorated, with a coma-like disturbance of consciousness, rapid extension of necrosis throughout the right front-parietal-temporal region (Fig. 1B), and multi-visceral failure.

**Fig. 2:**
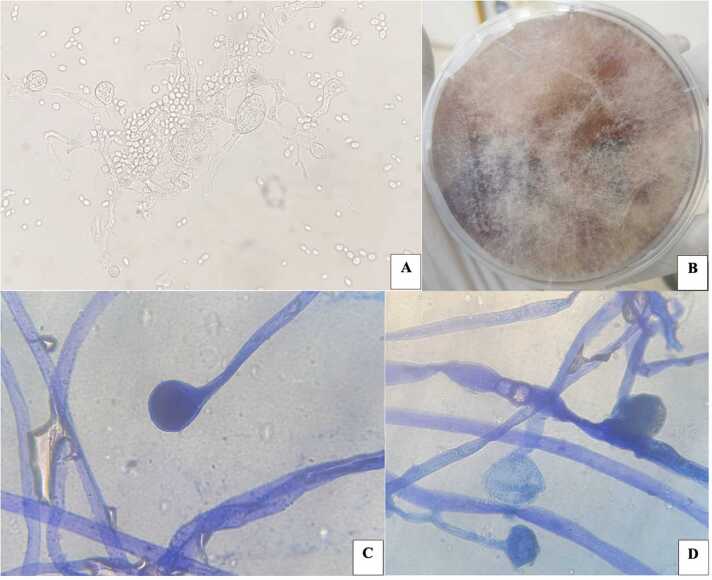
A) Direct microscopic examination of the sample showing irregular and branched hyphae forming a columella. B) Extensive and white cottony colonies of *Rhizopus oryzae* isolated on Sabouraud-Dextrose-Agar. C-D) Microscopic aspect of *Rhizopus oryzae* visualised with blue lactophenol showing the same columella and sporangia filled with sporangiospores (D).

A cerebral CT scan was requested, and a microbiological analysis of skin lesions was ordered on the dermatologist's advice. The cerebral CT scan showed chronic pansinusitis, which is more advanced at the ethmoidal level, with diffuse cerebral hypodensity and edematous cortico-subcortical differentiation without collection. Direct microscopic examination of the skin sample showed the presence of budding yeasts and broad, irregular, branched, and non-septate hyphae, some of which formed columella associated with numerous spores (Fig. 2A). This led to the inoculation on Sabouraud-Dextrose Agar (SDA) added with chloramphenicol. After two days (day 3), appeared on SDA, very fast-growing, extensive and white cottony colonies of a mold (Fig. 2B). Microscopic examination on blue lactophenol mount revealed sporangia showing sporangiospores (Fig. 2C-D), which were identified as *Rhizopus* sp. in collaboration with the Parasitology and Mycology laboratory at Le Dantec University Hospital. The diagnosis of mucormycosis in diabetic ketoacidosis was accepted. The patient died on the third day of hospitalisation before a definitive diagnosis could be made. Furthermore, the isolate and a blood sample were sent to the Laboratory of Parasitology and Mycology at IHU in Marseille, France, for confirmation. In-house *Mucorales* real-time qPCR on the blood sample was negative. At the same time, the strain was confirmed as *Rhizopus oryzae* by a Matrix Assisted Laser Desorption/Ionisation-Time Of Flight Mass Spectrometry (MALDI-TOF MS).

## Discussion

Rhino-orbital-cerebral mucormycosis is a rare but rapidly progressive invasive fungal infection with a worldwide distribution. India is recognised as the country most affected by diabetes and mucormycosis in the world, mostly during the COVID-19 pandemic [8]. This fungal infection is rarely reported in Africa, with most cases occurring in the Maghreb. Very few cases have been reported in sub-Saharan Africa, including one case in Senegal, dating back over half a century [9]. The analysis of these cases reveals diagnostic shortcomings in some cases, underlining the challenge it may represent. While some have relied solely on a biopsy to make the diagnosis [10], others have described long evolutionary durations over the years with no identified risk factor and no mycological evidence [2], [7], [11]. These diagnostic difficulties may be attributable to a lack of clinical-biological collaboration. A survey of clinicians' knowledge and experience of fungal infections in Senegal showed that clinicians do not often think of invasive fungal infections, and those who suspect these conditions do not use mycology to support their suspicion, relying, for the most part, solely on clinical diagnosis [12]. It may also be related to the unavailability of medical mycology laboratories because, in this case, the hospital where the patient was admitted did not have a proper medical mycology laboratory. Yet, positive direct examination is a sign of mycosis and often enables specific treatment to be started [13]. In this case, a well-informed eye would at least allow the diagnosis of mucormycosis before isolation and precise identification of the species involved. However, the technician who received and handled the sample did not appreciate the importance of what he had visualised, pointing out above all the presence of yeasts. The direct examination was repeated the next day after fungal growth. This also raises the need to upgrade the technical capacities of the national laboratories, which only rely on conventional techniques, as molecular biology techniques have proven their efficiency and improved diagnostic accuracy. Indeed, metagenomics next-generation sequencing was used to confirm *R. oryzae* in ROCM in China by [1]. The death of the patient recorded four days after the hospitalisation underscores the importance of early diagnosis and treatment due to the rapid progression of the infection, and its potential fatality. Unfortunately, the unavailability of systemic antifungals is another problem in Africa. While some of these drugs are available in North Africa, this is not yet the same situation in sub-Saharan Africa, particularly in the West African sub-region, where fluconazole is very often the only systemic antifungal available, sometimes together with itraconazole in some countries [14]. On the other hand, fluconazole and voriconazole have no *in vitro* activity on *Mucorales*, while itraconazole is not experimentally effective in animals infected with *Rhizopus* sp. [15]. Amphotericin B is the best available antifungal against *Mucorales*, as demonstrated by the successful outcome of the various cases in which it has been used [2], [11], [16], [17].

The fatal outcome of this case and the errors noted at various levels of the management chain should serve as lessons to avoid similar situations in the future. However, the limitations of this study are the professionals' lack of experience and consideration of invasive fungal infections. The latter are often ignored or underestimated in comparison with major communicable diseases such as HIV infection, tuberculosis or malaria [18]. This case is a typical example of an infection that requires multidisciplinary management, including ENT, ophthalmology, neurology, infectious diseases, intensive care, imaging, surgery and so on. Above all, close clinical-biological communication is essential, given the rapid evolution of the clinical picture. However, even if these conditions were met, the availability of essential antifungal molecules such as amphotericin B is a *sine qua non* condition for ensuring the patient's vital prognosis. Therefore, the health authorities are called upon to align themselves with international guidelines, notably those recommended by the World Health Organisation (WHO). The WHO has published a report presenting the first list of priority fungal pathogens [19]. This report of 19 fungi that represent the greatest threat to public health includes the *Mucorales* involved in this case report. Likewise, the WHO periodically publishes a list of essential drugs estimated as minimum medical needs for a basic health care system, and the 2019 version, the last one, included amphotericin B, flucytosine, itraconazole and voriconazole [20]. Nevertheless, none of these molecules is available in Senegal.

## Conclusion

Although our patient presented with a classic clinical form of mucormycosis, both clinical and biological diagnoses could not be made in time. This highlights the need for training at all levels in the knowledge, recognition and appropriate treatment of fungal infections, particularly invasive ones. Similarly, the availability of molecular tools in laboratories, as well as systemic antifungal agents, is a complementary condition for rapid and efficient treatment. In the meantime, the creation of a comprehensive registry to collect standardised data could improve our understanding of the epidemiology of invasive fungal infections, especially mucormycosis in sub-Saharan Africa, as in the absence of rapid diagnosis and management, rhino-orbital-cerebral mucormycosis is always rapidly fatal.

## CRediT authorship contribution statement

**Khadim Diongue:** Writing – original draft, Methodology, Conceptualization. **Abdourakhmane Sané:** Investigation. **Sokhna Moumi Daffé:** Writing – review & editing, Investigation, Data curation. **Stéphane Ranque:** Validation. **Papa Silman Diawara:** Methodology. **Bécaye Fall:** Supervision. **Mor Ngom:** Methodology. **Mamadou Gueye:** Methodology. **Maguette Ndoye:** Methodology.

## Consent

Informed consent was obtained from the patient's parents for publication of this case report.

## Ethical approval

This was approved by the internal ethics committee of the hospital.

## Funding

This research received no specific grant from any funding agency in the public, commercial or not-for-profit sectors.

## Conflict of interest

There are none.

## Declaration of Competing Interest

The authors declare that they have no known competing financial interests or personal relationships that could have appeared to influence the work reported in this paper.
